# Do Mesocarnivores Respond to the Seasonality in Management Practices in an Agroforestry Landscape?

**DOI:** 10.1007/s00267-024-02003-2

**Published:** 2024-06-08

**Authors:** Ana Luísa Barros, Diogo Raposo, João David Almeida, Sandra Alcobia, Maria Alexandra Oliveira, Darryl I. MacKenzie, Margarida Santos-Reis

**Affiliations:** 1 cE3c - Centre for Ecology, Evolution and Environmental Changes & CHANGE - Global Change and Sustainability Institute, Faculdade de Ciências da Universidade de Lisboa, Campo Grande, Lisboa, Portugal; 2 Proteus Research and Consulting Ltd, P.O. Box 7, Outram, New Zealand

**Keywords:** Camera-trap, High Nature Value Farmland, Mesocarnivore, Multi-season occupancy, Oak woodland

## Abstract

In the Mediterranean, we find a mosaic of natural and cultural landscapes, where a variety of forest management practices created intermediate disturbance regimes that potentially increased biodiversity values. Nonetheless, it is essential to understand the species’ long-term response to the dynamic management in agroecosystems, since the species tolerance to disturbance can change throughout the life cycle. Mammalian carnivores can be sensitive to human disturbance and are an essential part of ecosystems due to their regulatory and community structuring effects. We investigated the spatial response of five mesocarnivores species to spatially- and temporally- varying management practices in an agroforestry landscape. More specifically, we assessed the mesocarnivores’ temporal changes in space use by implementing multi-season occupancy models in a Bayesian framework, using seasonal camera-trapping surveys for a 2-year period. All species had a weak response of local extinction to forestry management and livestock grazing pressure. For forest-dwelling species, occupancy was higher where productivity of perennial vegetation was high, while colonization between seasons was positively associated with vegetation cover. For habitat generalist species, we found that occupancy in the wet season increased with the distance to cattle exclusion plots. Most of these plots are pine stands which are subject to forestry interventions during winter. During the 2-year period we found seasonal fluctuations in occupancy for all species, with an overall slight decrease for three mesocarnivore species, while for the two forest-dwelling species there was an increase in occupancy between years. The weak species response to management practices supports the importance of traditional management for upholding a diverse mesocarnivore community in agroforestry systems but could also reflect these species’ ecological plasticity and resilience to disturbance.

## Introduction

European landscapes have been shaped by centuries of human activities, particularly by the conversion of land into pastures and croplands. In 2020, agricultural landscapes were estimated to cover 39% of the total land area of the European Union, which is slightly above the world average (35%; Eurostat 2023). Traditional farming systems, characterized as low-input/low-output systems, usually hold high biodiversity values and are commonly known as High Nature Value Farmland (Bignal and McCracken 2000; EEA 2004). The Mediterranean basin is classified as a biodiversity hotspot (Myers et al. 2000), where HNVF systems are still preserved (Bignal and McCracken 2000). Through a variety of forest management practices (e.g., controlled burning, livestock husbandry, water management), sustainable agro-silvo-pastoral ecosystems were established, where intermediate disturbance regimes (Blondel 2006) have potentially increased biodiversity values (Benton et al. 2003; Bugalho et al. 2011; Moi et al. 2020). The *montado* (or *dehesa* in Spanish) is a cultural agroforestry landscape common throughout the Mediterranean region. This cork oak (*Quercus suber*) and/or holm oak (*Quercus rotundifolia*) woodland resulted from the gradual and selective thinning and shrub clearing of the original and dense evergreen oak forest (Sá-Sousa 2014), creating a savannah-like landscape. Here, forestry coexists with livestock raising, a main economic resource in these systems, along with the extraction of cork and timber (Blondel 2006). The rotation of crops with fallow/pastures and livestock transhumance with low stocking numbers characterized most *montado* landscapes, but these practices are in decline (Bugalho et al. 2011). Livestock plays an important role in controlling shrub encroachment, which otherwise is done through mechanical methods. In less accessible areas, dense shrub patches are maintained (Blondel 2006). The combination of these different management options creates a multifunctional landscape.

Species adapted to the diversity of structures and resources found in HNVF are vulnerable under high-intensity management (Henle et al. 2008; Rockstrom et al. 2009). Several studies have demonstrated the importance of landscape heterogeneity for different taxonomic groups (Holland and Fahrig 2000; Weibull et al. 2000; Curveira-Santos et al. 2017; Benedek and Sîrbu 2018) but a better understanding of the direct impacts from alternative management options is essential to uphold both the economic and biodiversity targets in these mosaic agroforestry systems (Curveira-Santos et al. 2021). Furthermore, the same dynamic management that creates such heterogenous and rich landscapes also requires long-term monitoring of its effects, as species’ ecological requirements and tolerance to disturbance can also change throughout their life cycle (Benton et al. 2003; Monterroso et al. 2014; Shamoon et al. 2017). Mammalian carnivores play an important role in regulating ecosystems (Ripple et al. 2014) and drive community structure through trophic cascades (Roemer et al. 2009). Therefore, they are essential for ecosystem functioning. In many Mediterranean landscapes, mesocarnivores are the remaining species ensuring predation functions in the absence of larger predators (Temple and Cuttelod 2009), but also several other complex ecological roles (e.g., seed dispersing, nutrient subsidies) due to the diversity of life-history traits and behaviors of this taxonomically diverse group (Roemer et al. 2009). Generally, mesocarnivores have a higher tolerance to human disturbance than larger carnivores (Crooks 2002; Parsons et al. 2018), higher population densities and broader trophic niches (Rosalino and Santos-Reis 2009). In Mediterranean agroecosystems, the mesocarnivore assemblage can be quite rich and includes species with a wide range of ecological requirements. From abundant habitat generalists (e.g., red fox, *Vulpes vulpes*, and Egyptian mongoose, *Herpestes ichneumon*) to more specialist and rare species (e.g., western polecat, *Mustela putorius*, wildcat, *Felis silvestris*) and several others in between (e.g., common genet, *Genetta genetta*, stone marten, *Martes foina*) (Barrull et al. 2014; Soto and Palomares 2015). Thus, the mesocarnivores’ response to agroforestry management can be as diverse as the carnivore guild itself (Pita et al. 2009; Curveira-Santos et al. 2017; Shamoon et al. 2017).

In this paper, we investigated the species’ spatial response to spatially- and temporally- varying management practices in a sustainably managed *montado* landscape characterized by permanent human disturbance and seasonality in agroforestry practices. More specifically, we used camera-trapping surveys during a 2-year period to assess the mesocarnivores’ seasonal extinction and colonization patterns, based on changes in space use from occupancy estimates. Given the different ecological requirements of the species, in terms of habitat structure we expected (i) a stronger response from forest-dwelling species, such as the genet and the stone marten, with occupancy and colonization probabilities being mediated by the availability of vegetation cover, especially shrubs and trees (Santos-Reis et al. 2005). Conversely, (ii) habitat generalist species like the red fox, the Eurasian badger (*Meles meles*), and the Egyptian mongoose, are expected to have weaker associations with vegetation cover (Curveira-Santos et al. 2017). In terms of the response to disturbance, we expected the (iii) genet and the stone marten to respond negatively to the direct disturbance from forestry practices and cattle grazing pressure given their impact on vegetation cover (Gonçalves et al. 2011). Conversely, the (iv) badger could benefit from cattle presence as it increases arthropod availability, a main trophic resource for this species (Hipólito et al. 2016). Overall, (v) we expect seasonal but not yearly fluctuations in species occupancy.

## Methods

### Study Area

The study was conducted at Companhia das Lezirias (CL; 38°50′32.6″N, 8°49′56.5″W), the largest agroforestry farmstead in Portugal (~180 km^2^) and a research and monitoring site of a Long-Term Socio-Ecological Research Platform (LTSER Montado). Nearly 66 km^2^ of the estate is cork oak *montado* interspersed by patches of pine stands, scrublands, and agricultural land uses. Forestry management at CL has been internationally certified as sustainable since 2010 (certificate SA – FM / COC – 002659), and the estate is partly classified as a Special Protection Area (PTZPE0010) and Site of Community Importance (PTCON009) under the Natura 2000 network. The *montado* has a variable composition and density of understory, depending on grazing pressures and/or shrub clearance activities. Cattle are raised in a rotating system between grazing plots, with some areas permanently excluded from grazing. From late September until February/March, cow herds rotate among plots and in the spring, the cattle are guided to the estate’s marshy areas (Gonçalves et al. 2011). The climate is Mediterranean and between 2020 and 2022, the mean temperature was 17.6 °C and the annual rainfall was 338 mm. The study area has a diverse mesocarnivore community, with eight out of the ten species occurring in Portuguese *montado* (Gonçalves et al. 2011; Bencatel et al. 2018). Of these, five species are more abundant and were the target of this study. The red fox, the Eurasian badger and the Egyptian mongoose are characterized as habitat generalists, and the common genet and the stone marten as forest-dwelling species.

### Mesocarnivore Surveys

**Fig. 1:**
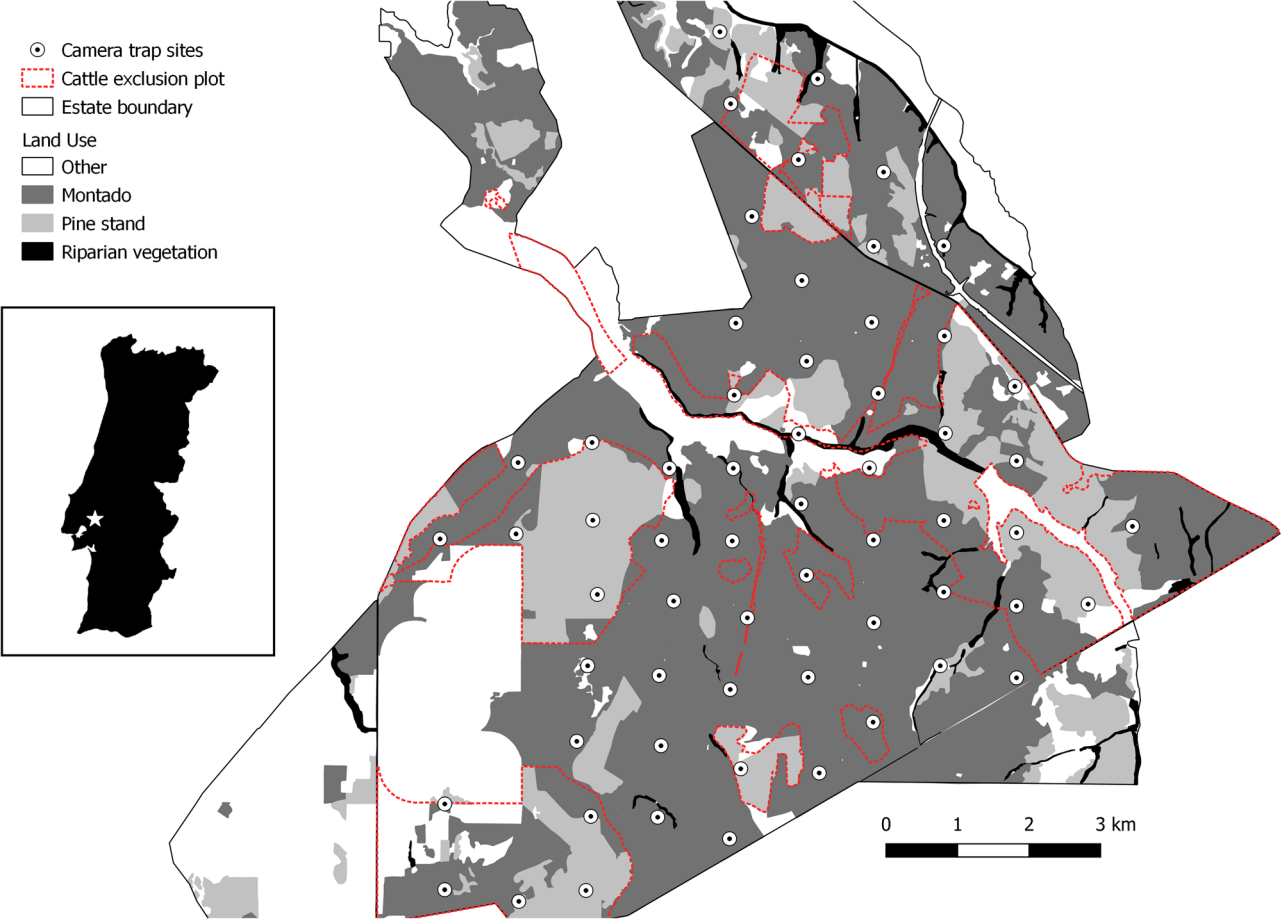
Location of camera trap sites in the study area (Companhia das Lezírias) and the most relevant land uses. During each of the four seasonal surveys, camera traps were deployed at 60 locations distanced 1035 m (on average) from each other. On the left panel there is a map of Portugal with the study area’s location marked with a white star

From 2020 to 2022, camera traps were deployed at the study area for surveys in the wet (October to December) and dry (June to August) seasons. In each season, we surveyed 60 camera trap sites, defined as the centroid of 1 km grid cells (Fig. 1). After slight adjustments in the field, the average distance between sites was 1035 m (SD = 76 m, min = 864 m, max = 1261 m). Camera traps were mounted on trees 20–30 cm above the ground (Kelly 2008), and paths, trails, or other landscape structures were not preferentially targeted but selected when available at a location. No bait or lure was used near the camera traps. During the wet season, all camera traps were Browning Dark Ops HD Pro X (trigger speed of 0.22 sec), set to take three photographs per trigger with a 5-s interval. In the dry season, cameras were Cuddelink Long Range IR model J-1521 (trigger speed of 0.25 s), set to take three photographs per trigger with a 1-s delay between bursts. Each Cuddelink camera was connected to a solar panel but also had alkaline batteries in case the panel malfunctioned. We used different camera models between seasons because of camera failures of the Browning model at high temperatures during the dry season. Sampling effort resulted in the following trapping days per season: 3941 and 3474 days in the wet seasons of 2020 and 2021 (hereafter coded as W20 and W21), respectively, and 4262 and 3800 days in the dry seasons of 2021 and 2022 (hereafter coded as D21 and D22), respectively.

### Multi-Season Occupancy Models

Occupancy models are hierarchical models that account for species’ imperfect detection as this can cause the underestimation of the probability of a species occupying a site (i.e., occupancy probability) due to “false absences” in the data set. Through repeated surveys of a site within a sampling period, the detection probability of a species ($p$) can be estimated and used to correct the occupancy probability estimate ($\varphi$) (MacKenzie et al. 2018). A key assumption of these models is that the occupancy state of a site does not change during a sampling period. For estimating occupancy across sampling periods (e.g., seasons or years), not accounting for imperfect detection can further bias estimates of change in occupancy (MacKenzie et al. 2003). Thus, multi-season occupancy models are more suited for long-term studies, especially when focusing on the mechanisms governing site occupancy dynamics, as it enables to also estimate local colonization and extinction probabilities (MacKenzie et al. 2003). Local colonization (${\gamma }_{t}$) refers to the probability that a site unoccupied at a primary period *t* is occupied by the species at *t* + 1; and extinction (${\varepsilon }_{t}$) is the probability of a site occupied by the species at *t* is unoccupied at *t* + 1. These are dynamic processes that represent the probabilities of a site transitioning between the occupied and unoccupied state between seasons (MacKenzie et al. 2018) and in our study, these parameters indicate seasonal changes in space use rather than population extinction/colonization processes. Also, we used the original parameterization from MacKenzie et al. (2003) where occupancy is estimated directly for the first sampling period (hereafter, first-season occupancy that refers to W20) along with colonization and extinction probabilities, while occupancy in subsequent periods is derived from these values.

In multi-season occupancy models, *N* sites are surveyed over time during *T* primary sampling periods, between which changes in site occupancy state may occur. Within each primary period, detection/non-detection surveys are conducted on *k* occasions or secondary sampling periods. This allows building a detection history for each site expressed as *T* vectors, where 1 denotes a species as detected and 0 as non-detected for each secondary period. As detection events of the mesocarnivore species are collected in continuous time, we discretized the observations in each season in consecutive 5-day occasions (i.e., secondary periods) where, if a species was detected during that 5-day period, this was summarized as a 1 (=detected) in that occasion, or zero otherwise. Overall, each season (i.e., primary period) was composed of 13 secondary periods.

### Environmental Predictors

We selected a set of variables related to habitat structure and disturbance from agroforestry practices to model the state (i.e., first-season occupancy, local extinction and colonization) and observation (i.e., detection) parameters. Habitat variables were mainly related to the availability of vegetation cover, which is typically used for refuge or foraging, but also reflect the forestry practices implemented. Additionally, we defined disturbance variables related to the main anthropogenic activities in these landscapes that can potentially influence species behavior (i.e., space use). Site-specific variables can be either constant (hereafter site variables) or variable (hereafter season-site variables) between primary periods. These are used to model all parameters, while observation-specific variables that vary between secondary periods can only be used to model detection probabilities ($p$). Given these three types of variables, we defined four site variables related to habitat structure and composition to model species’ first-season occupancy ($\varphi$); four season-site variables, two related to direct disturbance from agroforestry practices and two related to vegetation cover availability, to model extinction ($\varepsilon$) and colonization ($\gamma$) probabilities, respectively; and, to model species detection ($p$), we defined three site variables and one observation variable (Table 1).

**Table 1:** Environmental predictors used to model the four parameters of multi-season occupancy models (first-season occupancy - $\varphi$; local extinction – ε; local colonization – γ; detection – p) for each of the five target mesocarnivore species in the study area

Variable code	Description	Units	Mean (SD)	Parameter
	*Site variables*			
D_Rip	Euclidian distance between a camera trap site and the nearest riparian vegetation patch (i.e., linear strips of dense vegetation surrounding streams and primarily composed of willows, ashes, alders and blackberries). Carnivores are expected to use these structures more often than the surrounding matrix.	meters	753 (664)	$\varphi$
D_Excl	Euclidian distance between a camera trap site and the nearest fenced plot where cattle is permanently excluded. Preservation of the vegetation structure in these plots should provide refuge, but certain prey items associated with cattle may be scarce.	meters	230 (303)	$\varphi$
MINV	Annual parameter of plant phenology and productivity corresponding to the average vegetation index value of minima on left and right sides of the vegetation productivity curve for a given season. This parameter represents the productivity of perennial vegetation, mainly the shrub and tree cover, that provide important shelter and food items. See more details on Online Resource A	PPI x day^−1^	0.08 (0.03)	$\varphi$
TPROD	Annual parameter of plant phenology and productivity corresponding to the total productivity in the first season. This parameter represents the productivity of both perennial and annual vegetation, both used for shelter and cover. See more details on Online Resource A	PPI x day^−1^	115 (18)	$\varphi$
	*Season-site variables*			
Graz	Cattle grazing pressure, given by the number of days (d) in a camera trapping season, is the number of livestock units (*LSU*) in a plot of a given area (a). Calculated as: $\left(\frac{{LSU}}{a}\right)\times {d}$, for a 500 m radius buffer around the camera trap site. Typically associated with habitat disturbance.	$\frac{{LSU}}{a}\times {days}$	6.27 (13.24)	$\varepsilon$
ForInterv	Ratio of area in a 500 m radius buffer around the camera trap site where forestry work (e.g., logging, pruning, shrub clearing) was conducted before each camera trapping season. Removal of vegetation is expected to reduce refuge for carnivores.		0.14 (0.25)	$\varepsilon$
NDVI	Mean of the *Normalized Difference Vegetation Index* (NDVI) calculated for each 10 m pixel in a 500 m radius buffer around the camera trap site. Values range from −1 to 1. A decrease in vegetation availability between seasons can negatively impact carnivores. See more details on Online Resource A		0.53 (0.1)	$\gamma$
NDVIstdv	Mean of the standard deviation of NDVI values calculated for a 500 m radius buffer around each camera trap site. Measure of vegetation heterogeneity, which can favor some carnivore species.		0.12 (0.04)	$\gamma$
Season	Categorical covariate with 4 levels corresponding to each of the camera trapping surveys conducted between 2020–2022. Levels coded as *W20*, *D21*, *W21*, *D22* for each of the primary periods.		-	$p$
Shrubs	Percent of shrub cover visually estimated for a 50 m radius buffer around a camera trap site. Carnivores use vegetation cover to move in the landscape, but it can also affect camera trap performance.	%	39 (31)	$p$
Feat	Categorical covariate indicating the type of feature in a camera traps field of view. Either *trail*, *dirt road* or *none*. Carnivores tend to use features such as trails more often.		-	$p$
Alt	Categorical covariate indicating if vegetation in the camera trap’s field of view was cleared, as this can affect the species behavior and detection.		-	$p$
	*Observation variables*			
Effort	Number of days a camera trap was active in a 5-day occasion, with a minimum of 1 day for a camera to be considered as active, otherwise it was considered *NA*	days	4.86 (0.56)	$p$

Variables are divided into three types: site variables which are site specific, but constant across primary periods (i.e., seasons); season-site variables which vary between primary periods and sites; and observation variables which vary across primary and secondary (i.e., occasions) periods, and between sites. Each variable was attributed a code name and includes a description with measurement units

We assessed vegetation status using high spatial resolution remote sensing (RS) optical data derived from multispectral imagery from Sentinel 2. Data processing followed a two-fold approach, focusing on (i) time-aggregated phenology variables and (ii) RS vegetation parameters overlapping each survey season. Using the High-Resolution Vegetation Phenology and Productivity (HR-VPP) product from Copernicus we selected two of the 13 parameters (Table 1) that depict different stages of the vegetation growth curve (Smets et al. 2020) to model first-season occupancy ($\varphi$). Also, we used the Normalized Difference Vegetation Index (NDVI) determined from bottom of atmosphere reflectance level 2, Sentinel 2 images (Copernicus Sentinel data from 2020–2022) to model species local colonization ($\gamma$) (Table 1). For further details on the RS methodology see Online Resource A. Site and season-site variables were measured for a 500 m radius buffer around each camera site to reflect the species’ preferences in terms of space use, given the target species’ maximum mean core area documented in the literature (Eurasian badger: 0.94 km^2^; Rosalino et al. 2004). To model detection ($p$), we used season-site variables related to micro-habitat features visually estimated for a 50 m radius around the camera trap sites, and an observation variable for survey effort (Table 1). Continuous covariates were standardized (mean = 0 and SD = 1) before model fitting.

### Statistical Analysis

The modeling procedure involved two stages. First, we modeled species detection ($p$) by combining the effect of season with one other detection variable (see Table 1) to account for both the species’ seasonal behavioral changes and the use of two different camera models. Then we proceeded with model ranking (more details below) to select the covariate which best modeled species detection. Secondly, we maintained the covariates from the top-ranking model for detection probability and modeled species first-season occupancy ($\varphi$), local extinction ($\varepsilon$) and colonization ($\gamma$) probabilities as a function of variables. The candidate models were built to test specific hypotheses related to each species’ known ecological preferences from the literature (Santos-Reis et al. 2005; Hipólito et al. 2016; Curveira-Santos et al. 2017). To model these state parameters, we fit only univariate models, i.e., only one variable to model each parameter. We also opted to maintain either extinction or colonization constant when modeling either one of these parameters, i.e., we did not fit variables to model extinction and colonization simultaneously in a single model. We justify this by the low sample size (*N* = 60 sites) compared to the number of parameters estimated, which can exponentially grow for this set of occupancy models.

We fitted models in a Bayesian framework using STAN (Carpenter et al. 2017) called from R (version 4.1.3, R Core Team 2022) using the package ubms (version 1.2.2; Kellner et al. 2022). We generated three chains of 15,000 iterations each and discarded 5000 as burn-in. For the probability-type parameters ($\varphi ,\,\varepsilon ,\,\gamma ,{p}$) we chose the weakly informative priors Logistic(0,1) for intercepts and regression coefficients, and the prior Gamma(1,1) for the random effect standard deviations (Northrup and Gerber 2018). We assessed convergence by visually inspecting the trace plots and used the $\hat{R}$ statistic (Gelman and Rubin 1992), assuming no evidence of lack of convergence when $\hat{R}\,$< 1.1. For model ranking, we measured the predictive accuracy of each model through leave-one-out cross-validation (LOO-PSIS). Contrary to other more well-known criteria (e.g., AIC and DIC) that are based on point estimation, LOO is a truly Bayesian method for cross-validation (Vehtari et al. 2017). The models are ranked according to their expected log predictive density (elpd) value and the best model has the highest elpd value (Vehtari et al. 2017). We used pareto-$\hat{k}$ as a diagnostic of how far an individual leave-on-out distribution was from the full distribution. If $\hat{k}$ < 0.5, then the corresponding elpd was estimated with high accuracy; however, for $\hat{k}\, >\, 0.7$, the importance sampling was not able to provide useful estimates, and we decided to discard those models (Vehtari et al. 2017). If the elpd difference between candidate models is small (<4), predictive performance is similar, and we proceeded with model averaging. We used the approach from Yao et al. (2018) by stacking the combination of predictive distributions according to model weight to obtain the covariate effects on species occupancy and predict the mean occupancy for each season. We considered a covariate effect statistically significant when the 95% Bayesian credible interval (BCI) (2.5 and 97.5 percentiles) did not overlap zero.

## Results

The five target species were detected in all survey seasons, but the number of independent events (i.e., at least 30 min apart) for each species was much higher in the wet seasons (W20 = 926, W21 = 1100) compared to the dry seasons, in both years (D21 = 378, D22 = 162). Nonetheless, each species’ naïve occupancy (i.e., the proportion of sites where a species was detected at least once) was similar between years for the same season (Online Resource B: Table B1). Overall, the stone marten had the lowest naïve occupancy values (W20 = 27%, D21 = 12%, W21 = 30%, D22 = 7%) and the red fox had the highest values (W20 = 82%, D21 = 63%, W21 = 82%, D22 = 45%) in all sampling seasons. Due to the low number of capture events of genet and stone marten in the dry seasons, we opted to consider only the wet seasons in the multi-season occupancy models for these species (i.e., two primary periods), to avoid spurious results of a covariate effect on local colonization/extinction.

Species detection was best modeled by the combined effect of season and feature type for the red fox, badger, and genet (Online Resource B: Table B2), as detection was higher on dirt roads, followed by game trails. Also, fox and badger detections were higher in the wet seasons while genet detection was higher in the wet season of 2021 relative to 2020 (Online Resource B: Table B3). Mongoose’s detection was best modeled by the effect of season and survey effort (Table B2), as detection increased with survey effort and was also higher in the wet seasons (Table B3). For the stone marten, the top-ranking model did not include any covariates on detection (Table B2).

**Fig. 2:**
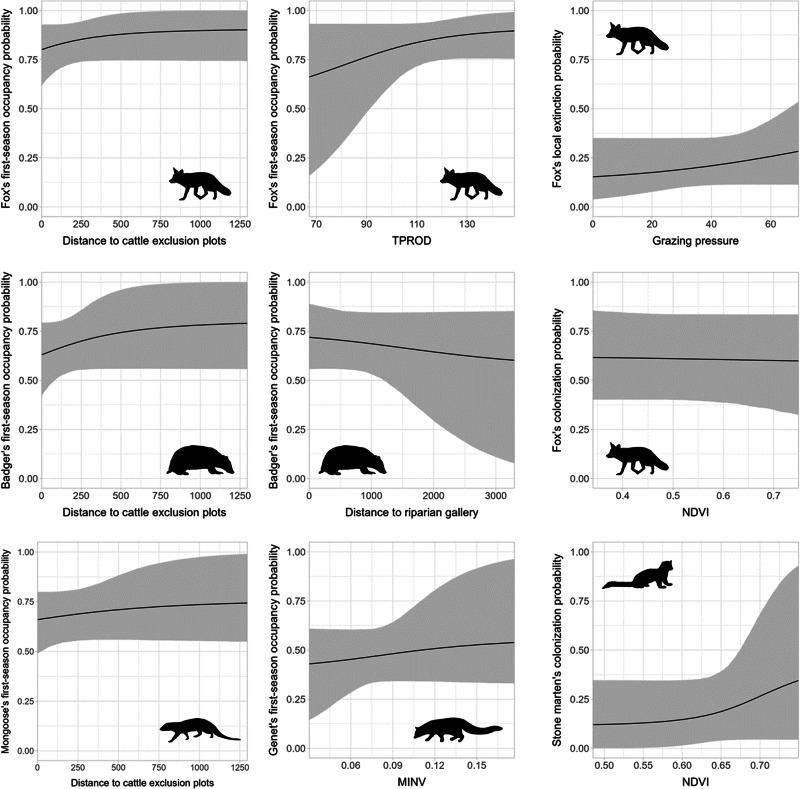
Covariate effect on state parameters $(\varphi ,\,\gamma ,\,\varepsilon )$ for each of the five mesocarnivore species detected at Companhia das Lezírias between 2020 and 2022. Effect plotted only for covariates with a significant marginal effect (BCI did not cross zero). Shaded area represents the 95% BCI. Red fox image from Anthony Caravaggi and used under license CC BY-NC-SA 3.0 DEED (https://creativecommons.org/licenses/by-nc-sa/3.0/). Images of the other species were dedicated to the public domain under license CC0 1.0 DEED

Ranking of the candidate models fitted with variables to model the state parameters did not reveal a single model with best predictive performance for any of the five species (Online Resource B: Table B4). Thus, we proceeded with model averaging to ascertain the covariate effect. For the red fox, total vegetation productivity (TPROD) and distance to cattle exclusion plots (D_Excl) had a positive effect on first-season (W20) occupancy, with occupancy increasing with vegetation productivity (coefficient = 0.51, SD: ± 0.38; Fig. 2) and the distance to exclusion plots (0.55, SD: ±0.55; Fig. 2). Extinction between seasons was best explained by cattle grazing pressure (Graz), as local extinction between seasons was lowest in plots of low grazing pressure (0.25, SD: ±0.31; Fig. 2), while colonization was lower in plots with high NDVI values (−0.03 ± 0.15; Fig. 2). Both effects were only marginally significant as the BCIs included zero (Table 2).

**Table 2:** Covariates’ beta coefficients from model averaging of candidate multi-season occupancy models with weight > 0, for each of the five mesocarnivores species detected at Companhia das Lezírias during seasonal surveys between 2020 and 2022

		Species				
Parameter	Covariate	Red fox	Badger	Mongoose	Genet	Stone marten
First-season occupancy	TPROD	0.51 (0; 1.35)	–	–	–	−0.46 (−1.31; 0.15)
	D_Excl	0.55 (0; 1.97)	0.41 (0; 1.9)	0.15 (0; 1.02)	–	
	D_Rip	–	−0.14 (−0.83; 0)	–	–	0.05 (−0.93; 0.42)
	MINV	–	–	–	0.11 (0; 0.9)	0.51 (−0.22; 1.85)
Extinction	Grazing	0.25 (0; 0.89)	−0.05 (−0.70; 0.11)	–	–	0.81 (−0.69; 4.42)
	ForInterv	–	–	–	–	−0.15 (−1.34; 1.44)
Colonization	NDVI	−0.03 (−0.49; 0)	–	0.02 (−0.73; 1.16)	0.26 (−0.11; 1.52)	0.62 (0; 3.08)
	NDVIstdv	–	–	0.08 (−0.53; 1.5)	–	

Values are presented only for covariates used to model first-season occupancy, colonization and local extinction, with the corresponding 95% BCI in parenthesis

Badger’s first-season occupancy was best modeled by distance to cattle exclusion plots (D_Excl) with a positive effect, and distance to riparian vegetation (D_Rip) with a negative effect. Therefore, badger occupancy probability in the first wet season (W20) increased with the distance to exclusion plots (0.41 ± 0.60; Fig. 2), while decreasing with the distance to riparian vegetation (−0.14 ± 0.24; Fig. 2). The effect of grazing pressure on local extinction was not statistically significant (Table 2).

First-season occupancy of mongoose was best modeled by the distance to cattle exclusion plots (D_Excl), as occupancy probability increased with the distance to those areas (0.15 ± 0.31; Fig. 2). From model averaging, no covariates were retained to model local extinction, and the colonization covariates (NDVI and NDVIstdv) did not have a statistically significant effect (Table 2).

For stone marten, the site covariates retained to model first-season occupancy were not statistically significant (Table 2), but the two vegetation productivity covariates had opposite effects on species occupancy (TPROD: −0.45 ± 0.4; MINV: 0.51 ± 0.55; see Online Resource B: Fig. B1). Stone marten’s colonization between wet seasons was slightly higher in plots with higher NDVI values (0.62 ± 0.97; Fig. 2). Genet’s first-season occupancy increased with MINV (0.11 ± 0.26; Fig. 2) and the only covariate retained after model averaging to model colonization did not have a statistically significant effect (Table 2).

**Fig. 3:**
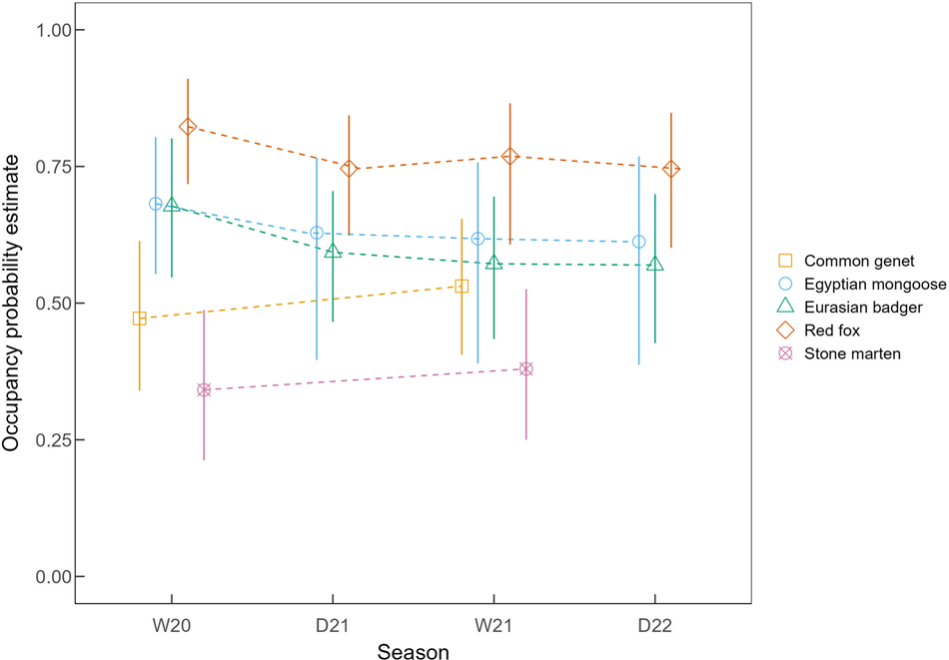
Seasonal occupancy estimates with 95% BCI (vertical lines) for each of the five mesocarnivore species detected at Companhia das Lezírias during the survey period of 2020–2022. Occupancy estimates were obtained from model averaging of the multi-season occupancy candidate model set. Seasons refer to each of the primary periods where camera trapping surveys were conducted, namely: wet season of 2020 (W20), dry season of 2021 (D21), wet season of 2021 (W21) and dry season of 2022 (D22)

Overall, occupancy estimates across seasons for the two survey years had only minor fluctuations (Fig. 3). Nonetheless, for the Eurasian badger and Egyptian mongoose, there was a slight decrease over time. For the badger, the decrease was 11% between wet seasons and 2% between dry seasons; for the mongoose, the decrease in occupancy was 6% between wet seasons and 2% between dry seasons. Contrastingly, common genet and stone marten’s occupancy slightly increased by 6% and 4%, respectively, between the two years. For the red fox, occupancy was lower during the dry seasons but recovered in the subsequent wet seasons. Despite this, occupancy between wet seasons still decreased by 5%.

## Discussion

Mediterranean agroforestry landscapes are subject to climatic and anthropogenic pressures that create a heterogeneous and dynamic landscape. By conducting seasonal surveys for two years, we found weak statistical evidence of an effect from the environmental and disturbance covariates on the dynamic processes of change in species’ occupancy. This result supports the low impact of the management practices implemented in the study area but can also reflect the ecological plasticity of these species (Díaz-Ruiz et al. 2013; Monterroso et al. 2014). The small fluctuations in occupancy estimates across the 2-year period may further support the importance of the sustainable agroforestry management in the area, but a longer-term assessment is necessary to properly establish population trends and interpret the current pattern.

The agroforestry practices conducted in the study area, such as cattle grazing, logging and shrub clearing, did not have a strong effect on species occupancy dynamics, even for those most vulnerable to these disturbances, such as the genet and the stone marten (Torre et al. 2022), contrary to our hypotheses. Nonetheless, stone marten’s colonization probability was correlated positively to NDVI, which is in accordance with the species’ preference for dense vegetation cover for resting (Santos-Reis et al. 2005). Although this covariate was not statistically significant for other species, models that account for this variable had some weight and, after model averaging, NDVI had a positive effect on badger, mongoose, and genet colonization probabilities. Particularly dense shrub patches have been shown as important habitat features for mesocarnivores in Mediterranean landscapes (Suárez-Tangil and Rodríguez 2023), both for shelter and food (Mangas et al. 2008; Gonçalves et al. 2011). Only the colonization of red fox between seasons was negatively associated with seasonal variations of NDVI, although with a very small effect size. As a habitat generalist, foxes have been shown to use open landscapes and agricultural matrix much more often than other mesocarnivore species (Virgós et al. 2002) and thus higher availability of vegetation cover might not be the main driver of local colonization. Contrary to expectations, only the local extinction of red fox was slightly influenced by grazing pressure, with extinction probability being lower in plots where grazing pressure was low in the previous season. Given that cattle herds are moved out of the study area during summer, this also means that extinction was lower between the dry and wet seasons. Therefore, cattle transhumance seems an important practice for the maintenance of stable carnivore populations. The negative impact of livestock on red foxes has been previously demonstrated in Mediterranean landscapes (Mangas and Rodríguez-Estival 2010; Curveira-Santos et al. 2017). This could be related to the decrease of shrub cover through grazing, which reduces small mammal richness and abundance (Gonçalves et al. 2011), an important food resource for the red fox (Díaz-Ruiz et al. 2013). Alternatively, low to medium grazing pressures and the rotation of herds between plots increase the availability of ground beetles (da Silva et al. 2008). This is a main food resource for species such as the badger, the stone marten, and the genet (Santos-Reis et al. 2005; Hipólito et al. 2016), and the low grazing pressure in the study period (average of ~13 and ~11 LSU in each winter season) (Almeida et al. 2016) might have benefited these mesocarnivores by providing such prey items.

For three species (red fox, badger, and mongoose), occupancy in the first winter season was positively associated with the distance to cattle exclusion plots. Some of these areas are *montado* plots which have been set aside since 2008, but several others are pine stands. Most forestry activities (i.e., logging and shrub clearing) are conducted in these pine stands during winter, which could lead species to avoid them during those periods. Thus, the avoidance of cattle exclusion plots might be partly correlated with the avoidance of pine plantations, which tend to be less suitable (Virgós et al. 2002). Additionally, the availability of coprophagous beetles is higher in grazing plots (Galante and Cartagena 1999) and this constitutes an important trophic resource for these mesocarnivores (Wierzbowska and Szalski 2010; Hipólito et al. 2016). Nonetheless, a better understanding of habitat structure and resource availability in these plots might give clearer insights into this result. Despite carnivore space use and richness being often associated with riparian habitat (Virgós 2001), this covariate was retained only for badger, with a weak correlation to first-season occupancy probability. In Mediterranean landscapes, these habitats are used as movement corridors and food sources (Rosalino et al. 2009); however, we expect a stronger association during summer when high temperatures drive animals in search of water, food, and cooler refuge (Santos et al. 2011). Red fox first-season occupancy was positively correlated with total vegetation productivity, which was used as a proxy for vegetation cover. These areas are often associated with high abundance of small rodents (Gonçalves et al. 2011) and are used for cover while moving across the landscape. For the two arboreal species (genet and stone marten), despite the low statistical support, minimum vegetation productivity (MINV) was a relevant covariate to model first-season occupancy. Since these species prefer forest habitats and use shrub and tree cavities to rest (Santos-Reis et al. 2005), this association was expected, although with a larger effect size. Nonetheless, we acknowledge the stone marten is characterized as a habitat generalist (Virgós and García 2002; Santos and Santos-Reis 2010) and its reliance on trees for resting is dependent on the availability of other structures and the presence of competitors and conspecifics (Santos-Reis et al. 2005). Furthermore, this covariate had a low variability in the study area (0.08, SD: 0.03), possibly related to the homogeneous distribution of oak trees, while the availability of shrub cover might be underestimated.

We acknowledge that the lack of strong statistical evidence of an effect (i.e., large effect size and narrow BCI) of the covariates could be due to sample size. By approximating the inter-camera distance to the core area size of the target species we were only able to define 60 sites in the study area. Increasing spatial replication should improve the ability to identify potential effects with higher confidence. Despite the complexity of this analytical framework, we consider that multi-season occupancy modeling is an appropriate tool to investigate the environmental drivers of occupancy change and the underlying processes (MacKenzie et al. 2018), while accounting for imperfect species detection which may bias estimates otherwise. Especially in disturbed and managed landscapes, directly modeling the processes governing these changes is essential to assess the impact of management actions and inform future management. Additionally, these models allow estimating trends in occupancy that are important for wildlife management (MacKenzie 2005; Smith et al. 2017; Sereno-Cadierno et al. 2023). Since our study covered only two years of surveys we could not establish long-term trends, although we found slight reductions in occupancy for the red fox, badger, and mongoose. One possibility for this slight decline in occupancy is the low rabbit availability in the study area, as this is a main prey of red fox and mongoose (Fernandez-de-Simon et al. 2015) and was previously associated with the occupancy of these two species in the study area (Curveira-Santos et al. 2017). Since then, rabbit populations have been declining, and in our study, the species was detected only at five camera trap sites during the two years. Contrastingly, stone marten increased occupancy between the two years, which seems part of a larger trend since in 2013–2014 there were only very few detections in this area (Curveira-Santos et al. 2017). Thus, the species might be recovering in the study area but this needs to be properly investigated. Therefore, it is essential to maintain monitoring efforts by extending the analysis for more survey seasons/years, and possibly by including other environmental predictors that were not tested here. Lastly, shifts in occupancy and habitat use are just one possible response of species to anthropogenic disturbance, which can also affect species population size and density (Lewis et al. 2015), vital rates (Pereira and Novaro 2014), home range size and dispersal (Main et al. 2020). Moreover, species might use other mechanisms to cope with disturbance, from adjustments in diel activity (Galvez et al. 2021) to changes in the use of food resources (Manlick and Pauli 2020).

## Conclusions

Traditional and sustainable agroforestry systems have been shown to support biodiversity values (Bignal and McCracken 2000) and carnivore richness in particular (Pita et al. 2009; Rosalino et al. 2009; Linck et al. 2023). Despite the dynamic management of the study area we found weak support for the influence of the current practices on mesocarnivore seasonal space use during the two year period. Although this result could be related to the species’ resilience to disturbance (Virgós et al. 2002), it also supports the value of sustainably managed agroforestry systems for biodiversity. Multi-season occupancy modeling provided a solid framework to explore hypothesis of the effect of agroforestry practices on the dynamic processes of occupancy change. By extending this approach over a longer time period we could potentially more accurately assess the impacts from current management and advise on future actions. Additionally, when surveying a smaller number of sites, a longer time period (i.e., four or more seasons/years) is necessary to obtain precise information on the occupancy trend (MacKenzie 2005). Therefore, maintaining monitoring efforts in the study area is essential to frame the current results and validate the coexistence between carnivores and agroforestry practices.

## Supplementary information

Supplementary Information

## Data Availability

The data used to fit the occupancy models will be provided upon request to the corresponding author.
