# Supplementary material for: Do Mesocarnivores Respond to the Seasonality in Management Practices in an Agroforestry Landscape?
Source: Environ Manage. 2024 Jun 8;74(4):636–47. doi: 10.1007/s00267-024-02003-2 (PMC11392974; doi:10.1007/s00267-024-02003-2)
Supplement: Supplementary file 1 — Supplementary Information [file 267_2024_2003_MOESM1_ESM.pdf]

Supplementary Information

Do mesocarnivores respond to the seasonality in management practices in an agroforestry landscape?

Ana Luisa Barros*, Diogo Raposo, João David Almeida, Sandra Alcobia, Maria Alexandra Oliveira, Darryl I. MacKenzie, Margarida Santos-Reis

* corresponding author: albarros@ciencias.ulisboa.pt

Appendix A – Methodology for remote sensing variables

The annual cycle of plants during the growing season was described by the High-Resolution Vegetation Phenology and Productivity (HR-VPP) product from Copernicus (Smets et al., 2020). The HR-VPP includes annual data for 13 parameters (e.g., start, end and length of growing season, productivity, amplitude, etc., Fig. A1) depicting stages of the vegetation growth curve, up to two growing seasons (Smets et al., 2020). Vegetation phenology was built on Sentinel 2 top of atmosphere level 1C multispectral images (Smets et al., 2021), namely the Plant Phenology Index (PPI) (Jin & Eklundg, 2014), inheriting the same spatial resolution of 10 m. Mosaic data was selected and extracted for the time-periods and overlapping the study area using the WEkEO portal. NDVI was computed for the study area using R statistical software (R Core Team, 2021), namely the sen2r package (Ranghetti et al., 2020). These indices and parameters were extracted at the plot level for a 500 m circular buffer around the camera trap sites. Data was spatially aggregated by determining weighted mean and standard deviation for each buffer using R statistical software, namely the raster package (Hijmans, 2021) and the sen2rts beta package (Ranghetti et al., 2021).

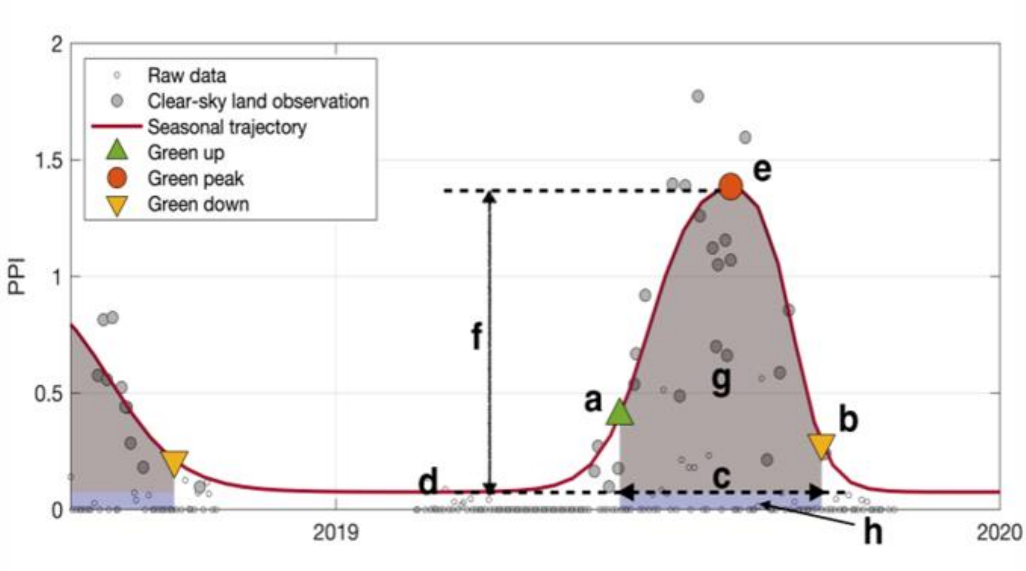

Fig. A1. Schematic representation of the Copernicus HR-VPP product. The parameters used to model species' first-season occupancy are represented by the letters d for MINV, and g+h for TPROD. Fig. from Smets et al. (2020).

References

Hijmans, R.J., 2021. raster: Geographic Data Analysis and Modeling. R package version 3.5-11. https://CRAN.R-project.org/package=raster

Jin, H., Eklundh, L., 2014. A physically based vegetation index for improved monitoring of plant phenology. Remote Sens Environ, 152, 512-525. https://doi.org/10.1016/j.rse.2014.07.010

R Core Team (2021). R: A language and environment for statistical computing.

Ranghetti L., Boschetti M., Nutini F., Busetto L., 2020. “sen2r: An R toolbox for automatically downloading and preprocessing Sentinel-2 satellite data.” Computers & Geosciences, 139, 104473. doi:10.1016/j.cageo.2020.104473

Ranghetti, L., 2021. “sen2rts: Build and Analyse Sentinel-2 Time Series”. R package version 0.4.0.9000. doi: 10.5281/zenodo.4682829,

Smets, B., Eklundh, L., Camacho, F., Jacobs, T., Van De Kerchove, R., Ivits, E., Zhanzhang, C., Tian, F., van Hoolst, R., Bonte, K., Sheifinger, G., Hufkens, K., Swinnen, E., 2020. Copernicus Land Monitoring Service-High Resolution Vegetation Phenology and Productivity (HR-VPP), Technical Specifications. Issue 1.1. Copernicus, European Environmental Agency, 37pp.

Smets, B., Cai, Z., Eklundh, L., Tian, F., Bonte, K., van Hoost, R., De Roo, B., Jacobs, T., Camacho, F., Sánchez-Zapero, J., Martinez-Sánchez, E., Swinnen, E., Scheifinger, H., Hufkens, K., Jönsson, P., 2021. High Resolution Vegetation Phenology and Productivity (HR-VPP) Seasonal Trajectories and VPP parameters User Manual. Copernicus Land Monitoring Service. European Environment Agency, 36pp.

Appendix B – Summary of mesocarnivore species detections, model selection and parameter estimates

Table B1. Summary of mesocarnivore species' independent events (i.e., at least 30 min apart) and naïve occupancy (i.e., percentage of sites where a species was detected at least once) for each of the seasons surveyed across the two years. The season code refers to the wet seasons of 2020 (W20) and 2021 (W21) and the dry seasons of 2021 (D21) and 2022 (D22).

	Capture events				Naïve occupancy (%)			
Species	W20	D21	W21	D22	W20	D21	W21	D22
Red fox	381	218	550	83	81.7	63.3	81.7	45
Eurasian badger	269	68	159	40	68.3	46.7	60	31.7
Egyptian mongoose	138	40	181	21	65	33	65	20
Common genet	100	43	169	14	43	30	51.7	21.6
Stone marten	38	9	41	4	26.7	11.7	30	6.7

Table B2. Model ranking of the candidate model set where species detection was modeled as a function of variables while occupancy, colonization and extinction were constant. Ranking was done through leave-one-out cross validation (LOO-PSIS) to assess each model's predictive accuracy. Models were ranked according to their expected log predictive density (elpd) value, with the top-ranking model having a higher elpd value. K indicates For all models, we tested the effect of a season-site or an observation variable combined with the effect of season, to account for seasonal behavioral changes and the two different camera trap models used. The covariates included in the top-ranking model (shaded grey) were then maintained in the next modelling stage.

Species	Models	elpd	K	Δelpd	ΔSE	weight
Red fox	$\varphi(.)\gamma(.)\varepsilon(.)p(Feat + Season)$	-1299.91	20.40	0	0	0.79
	$\varphi(.)\gamma(.)\varepsilon(.)p(Alt + Season)$	-1379.97	19.59	-80.06	25.74	0.03
	$\varphi(.)\gamma(.)\varepsilon(.)p(Effort + Season)$	-1382.70	19.26	-82.79	25.74	0.18
	$\varphi(.)\gamma(.)\varepsilon(.)p(Season)$	-1387.71	17.90	-87.80	26.26	0
	$\varphi(.)\gamma(.)\varepsilon(.)p(Shrubs + Season)$	-1391,19	23.66	-91.28	26.27	0
	$\varphi(.)\gamma(.)\varepsilon(.)p(.)$	-1435.97	8.67	-136.06	27.97	0
Badger	$\varphi(.)\gamma(.)\varepsilon(.)p(Feat + Season)$	-929.10	20.50	0	0	0.61
	$\varphi(.)\gamma(.)\varepsilon(.)p(Effort + Season)$	-950.95	15.83	-21.85	13.17	0.23
	$\varphi(.)\gamma(.)\varepsilon(.)p(Season)$	-951.86	15.21	-22.76	13.46	0
	$\varphi(.)\gamma(.)\varepsilon(.)p(Alt + Season)$	-952.43	16.89	-23.33	13.27	0
	$\varphi(.)\gamma(.)\varepsilon(.)p(Shrubs + Season)$	-952.45	19.12	-23.35	12.72	0
	$\varphi(.)\gamma(.)\varepsilon(.)p(.)$	-968.18	7.63	-39.08	19.52	0.16
Mongoose	$\varphi(.)\gamma(.)\varepsilon(.)p(Effort + Season)$	-826.82	13.81	0	0	0.76
	$\varphi(.)\gamma(.)\varepsilon(.)p(Season)$	-829.61	12.56	-2.79	3.28	0.12
	$\varphi(.)\gamma(.)\varepsilon(.)p(.)$	-858.66	7.22	-31.85	13.28	0.13
Genet	$\varphi(.)\gamma(.)\varepsilon(.)p(Feat + Season)$	-491.44	14.01	0	0	0.45
	$\varphi(.)\gamma(.)\varepsilon(.)p(Season)$	-492.55	9.44	-1.11	5.82	0
	$\varphi(.)\gamma(.)\varepsilon(.)p(Shrubs + Season)$	-493.21	12.13	-1.77	6.24	0.26
	$\varphi(.)\gamma(.)\varepsilon(.)p(Effort + Season)$	-493.48	10.75	-2.04	5.95	0
	$\varphi(.)\gamma(.)\varepsilon(.)p(Alt + Season)$	-494.28	11.74	-2.84	5.93	0
	$\varphi(.)\gamma(.)\varepsilon(.)p(.)$	-494.72	5.86	-3.28	7.18	0.29
Stone marten	$\varphi(.)\gamma(.)\varepsilon(.)p(.)$	-244.97	5.09	0	0	0.59
	$\varphi(.)\gamma(.)\varepsilon(.)p(Season)$	-246.33	6.7	-1.36	0.57	0
	$\varphi(.)\gamma(.)\varepsilon(.)p(Shrubs + Season)$	-246.38	8.01	-1.41	2.29	0
	$\varphi(.)\gamma(.)\varepsilon(.)p(Feat + Season)$	-246.52	10.52	-1.55	3.31	0.41
	$\varphi(.)\gamma(.)\varepsilon(.)p(Alt + Season)$	-246.9	7.44	-1.93	1.23	0
	$\varphi(.)\gamma(.)\varepsilon(.)p(Effort + Season)$	-247.42	7.74	-2.45	0.78	0

Table B3. Regression coefficients of the detection covariates from the top-ranking model for each mesocarnivores species detected during the camera-trapping surveys at Companhia das Lezírias between 2020 and 2020. The only exception was the stone marten since the top-ranking model did not include any variables to model detection. The coefficient estimates are presented with the corresponding 95% BCI in parenthesis.

Code	Intercept	Features		Season			Effort
Levels		Dirt road	Trail	D21	W21	D22	
Red fox	-1.26 (-1.49; 1.04)	1.57 (1.32; 1.82)	0.3 (0.03; 0.57)	-0.87 (-1.17; -0.58)	0.38 (0.13; 0.64)	-1.53 (-1.89; -1.17)	-
Badger	-1.53 (-1.84; -1.24)	1.07 (0.75; 1.41)	0.15 (-0.26; 0.56)	-0.82 (-1.23; -0.42)	-0.07 (-0.41; 0.27)	-1.22 (-1.71; -0.74)	-
Mongoose	-1.33 (-1.56; -1.1)	-	-	-1.38 (-1.84; -0.92)	0.32 (0.01; 0.63)	-1.86 (-2.44; -1.28)	0.19 (0.05; 0.34)
Genet	-1.71 (-2.12; -1.32)	0.63 (0.21; 1.06)	0.3 (-0.17; 0.77)	-	0.52 (0.16; 0.89)	-	-

* K refers to the effective number of parameters

* ΔSE is the standard error for elpd differences

* Weight is calculated through the stacking method to find the optimal model combination for maximizing the leave-one-out predictive density of the combination distribution. Used for model averaging

Table B4. Model ranking of the candidate model set that included variables to model each species state-parameters ($\varphi, \gamma, \epsilon$). Ranking was done through leave-one-out cross validation (LOO-PSIS) to assess each model's predictive accuracy. Models are ranked according to their expected log predictive density (elpd) value, however since the elpd difference between models was small, we proceeded with model averaging by stacking the combination of predictive distributions for models with weight > 0 (in shaded grey). Each species' detection was modeled using the covariates included in the top-ranking model in the previous stage (see Table B2).

Model structure	elpd	K	Δelpd	ΔSE	weight
**Red fox**					
$\varphi(D\_Exl)\gamma(.)\varepsilon(Graz)p(Feat + Season)$	-1297.40	22.01	0	0	0.02
$\varphi(TPROD)\gamma(.)\varepsilon(Graz)p(Feat + Season)$	-1297.59	22.68	-0.19	2.23	0.48
$\varphi(D\_Exl)\gamma(.)\varepsilon(.)p(Feat + Season)$	-1298.58	20.84	-1.19	2.41	0.37
$\varphi(TPROD)\gamma(.)\varepsilon(.)p(Feat + Season)$	-1298.70	21.45	-1.31	3.07	0
$\varphi(D\_Exl)\gamma(NDVI)\varepsilon(.)p(Feat + Season)$	-1298.84	21.67	-1.45	2.47	0.05
$\varphi(.)\gamma(.)\varepsilon(Graz)p(Feat + Season)$	-1298.90	21.75	-1.50	1.38	0
$\varphi(TPROD)\gamma(NDVI)\varepsilon(.)p(Feat + Season)$	-1298.95	22.18	-1.55	3.05	0
$\varphi(D\_Rip)\gamma(.)\varepsilon(Graz)p(Feat + Season)$	-1298.24	22.99	-1.85	1.74	0
$\varphi(.)\gamma(.)\varepsilon(.)p(Feat + Season)$	-1299.91	20.40	-2.51	2.69	0
$\varphi(.)\gamma(NDVI)\varepsilon(.)p(Feat + Season)$	-1300.05	21.14	-2.66	2.84	0
$\varphi(D\_Rip)\gamma(.)\varepsilon(.)p(Feat + Season)$	-1300.34	21.74	-2.94	2.85	0
$\varphi(D\_Rip)\gamma(NDVI)\varepsilon(.)p(Feat + Season)$	-1300.62	22.61	-3.22	2.84	0
$\varphi(.)\gamma(.)\varepsilon(.)p(.)$	-1435.97	8.67	-138.58	27.95	0.08
**Eurasian badger**					
$\varphi(D\_Exl)\gamma(.)\varepsilon(.)p(Feat + Season)$	-927.44	21.01	0	0	0.27
$\varphi(D\_Exl)\gamma(NDVI)\varepsilon(.)p(Feat + Season)$	-928.94	22.79	-1.50	0.28	0
$\varphi(D\_Exl)\gamma(.)\varepsilon(Graz)p(Feat + Season)$	-928.96	22.87	-1.53	1.12	0.14
$\varphi(.)\gamma(.)\varepsilon(.)p(Feat + Season)$	-929.10	20.50	-1.66	1.96	0.23
$\varphi(D\_Exl)\gamma(NDVIstdv)\varepsilon(.)p(Feat + Season)$	-929.24	23.33	-1.80	0.84	0
$\varphi(D\_Rip)\gamma(.)\varepsilon(.)p(Feat + Season)$	-929.27	21.63	-1.83	2.5	0.27
$\varphi(TPROD)\gamma(.)\varepsilon(.)p(Feat + Season)$	-930.19	21.59	-2.75	1.97	0
$\varphi(.)\gamma(NDVI)\varepsilon(.)p(Feat + Season)$	-930.48	22	-3.05	1.98	0
$\varphi(.)\gamma(.)\varepsilon(Graz)p(Feat + Season)$	-930.63	22.22	-3.19	2.19	0
$\varphi(D\_Rip)\gamma(NDVI)\varepsilon(.)p(Feat + Season)$	-930.64	23.09	-3.20	2.54	0
$\varphi(D\_Rip)\gamma(.)\varepsilon(Graz)p(Feat + Season)$	-930.85	23.46	-3.42	2.61	0
$\varphi(.)\gamma(NDVIstdv)\varepsilon(.)p(Feat + Season)$	-930.89	22.67	-3.45	2.08	0
$\varphi(D\_Rip)\gamma(NDVIstdv)\varepsilon(.)p(Feat + Season)$	-931.03	23.78	-3.59	2.6	0

$\varphi(TPROD)\gamma(NDVI)\varepsilon(.)p(Feat + Season)$	-931.59	23.09	-4.16	2	0
$\varphi(TPROD)\gamma(.)\varepsilon(Graz)p(Feat + Season)$	-931.74	23.37	-4.31	2.22	0
$\varphi(TPROD)\gamma(NDVIstdv)\varepsilon(.)p(Feat + Season)$	-932.10	23.89	-4.66	2.13	0
$\varphi(.)\gamma(.)\varepsilon(.)p(.)$	-968.18	7.63	19.18	19.18	0.09
**Egyptian mongoose**					
$\varphi(D\_Excl)\gamma(.)\varepsilon(.)p(Eff + Season)$	-826.74	14.55	0	0	0.2
$\varphi(.)\gamma(.)\varepsilon(.)p(Eff + Season)$	-826.82	13.81	-0.08	1.36	0.38
$\varphi(.)\gamma(NDVIstdv)\varepsilon(.)p(Eff + Season)$	-827.31	14.91	-0.57	1.54	0.02
$\varphi(D\_Excl)\gamma(NDVIstdv)\varepsilon(.)p(Eff + Season)$	-827.34	15.80	-0.61	1.2	0.11
$\varphi(.)\gamma(NDVI)\varepsilon(.)p(Eff + Season)$	-827.36	14.86	-0.63	1.5	0.2
$\varphi(D\_Excl)\gamma(NDVI)\varepsilon(.)p(Eff + Season)$	-827.47	15.96	-0.74	0.63	0
$\varphi(D\_Excl)\gamma(.)\varepsilon(Graz)p(Eff + Season)$	-827.69	15.81	-0.95	0.47	0
$\varphi(.)\gamma(.)\varepsilon(ForInterv)p(Eff + Season)$	-827.72	15.03	-0.98	1.45	0
$\varphi(.)\gamma(.)\varepsilon(Graz)p(Eff + Season)$	-827.73	15.02	-0.99	1.48	0
$\varphi(TPROD)\gamma(.)\varepsilon(.)p(Eff + Season)$	-827.80	14.98	-1.06	1.61	0
$\varphi(D\_Excl)\gamma(.)\varepsilon(ForInterv)p(Eff + Season)$	-827.85	16.08	-1.12	0.44	0
$\varphi(D\_Rip)\gamma(.)\varepsilon(.)p(Eff + Season)$	-828.02	15.44	-1.28	1.51	0
$\varphi(TPROD)\gamma(NDVI)\varepsilon(.)p(Eff + Season)$	-828.11	15.73	-1.37	1.70	0
$\varphi(TPROD)\gamma(NDVIstdv)\varepsilon(.)p(Eff + Season)$	-828.21	16.03	-1.47	1.76	0
$\varphi(TPROD)\gamma(.)\varepsilon(Graz)p(Eff + Season)$	-828.42	15.79	-1.68	1.7	0
$\varphi(D\_Rip)\gamma(NDVIstdv)\varepsilon(.)p(Eff + Season)$	-828.46	16.44	-1.72	1.7	0
$\varphi(D\_Rip)\gamma(NDVI)\varepsilon(.)p(Eff + Season)$	-828.52	16.45	-1.78	1.65	0
$\varphi(TPROD)\gamma(.)\varepsilon(ForInterv)p(Eff + Season)$	-828.65	16.06	-1.91	1.68	0
$\varphi(D\_Rip)\gamma(.)\varepsilon(ForInterv)p(Eff + Season)$	-828.86	16.51	-2.13	1.56	0
$\varphi(D\_Rip)\gamma(.)\varepsilon(Graz)p(Eff + Season)$	-828.91	16.60	-2.17	1.61	0
$\varphi(.)\gamma(.)\varepsilon(.)p(.)$	-858.66	7.22	-31.93	13.31	0.09
**Common genet**					
$\varphi(.)\gamma(.)\varepsilon(.)p(Feat + Season)$	-491.44	14.01	0	0	0.10
$\varphi(.)\gamma(NDVI)\varepsilon(.)p(Feat + Season)$	-491.58	15.05	-0.13	1.19	0.39
$\varphi(MINV)\gamma(.)\varepsilon(.)p(Feat + Season)$	-491.88	15.37	-0.43	1.66	0.25
$\varphi(MINV)\gamma(NDVI)\varepsilon(.)p(Feat + Season)$	-492.17	16.38	-0.73	1.68	0
$\varphi(.)\gamma(.)\varepsilon(Graz)p(Feat + Season)$	-492.39	15.13	-0.94	0.65	0
$\varphi(TPROD)\gamma(.)\varepsilon(.)p(Feat + Season)$	-492.43	15.02	-0.99	0.35	0
$\varphi(.)\gamma(.)\varepsilon(ForInterv)p(Feat + Season)$	-492.55	15.12	-1.10	0.85	0
$\varphi(TPROD)\gamma(NDVI)\varepsilon(.)p(Feat + Season)$	-492.60	16.10	-1.15	1.26	0

$\varphi(D\_Rip)\gamma(.)\varepsilon(.)p(Feat + Season)$	-492.65	15.26	-1.21	0.53	0
$\varphi(D\_Rip)\gamma(NDVI)\varepsilon(.)p(Feat + Season)$	-492.65	16.13	-1.21	1.33	0
$\varphi(MINV)\gamma(.)\varepsilon(Graz)p(Feat + Season)$	-492.85	16.50	-1.40	1.85	0
$\varphi(MINV)\gamma(.)\varepsilon(ForInterv)p(Feat + Season)$	-493.21	16.75	-1.77	1.80	0
$\varphi(TPROD)\gamma(.)\varepsilon(Graz)p(Feat + Season)$	-493.38	16.13	-1.93	0.72	0
$\varphi(D\_Rip)\gamma(.)\varepsilon(Graz)p(Feat + Season)$	-493.57	16.33	-2.13	0.84	0
$\varphi(TPROD)\gamma(.)\varepsilon(ForInterv)p(Feat + Season)$	-493.72	16.37	-2.28	0.98	0
$\varphi(D\_Rip)\gamma(.)\varepsilon(ForInterv)p(Feat + Season)$	-493.82	16.46	-2.38	1.00	0
$\varphi(.)\gamma(.)\varepsilon(.)p(.)$	-494.72	5.86	-3.28	7.18	0.26
**Stone marten**					
$\varphi(TPROD)\gamma(NDVI)\varepsilon(.)p(.)$	-241.69	6.71	0	0	0.39
$\varphi(MINV)\gamma(.)\varepsilon(Graz)p(.)$	-242.11	6.18	-0.42	3.23	0.25
$\varphi(MINV)\gamma(.)\varepsilon(ForInterv)p(.)$	-243.07	6.75	-1.38	3.00	0.15
$\varphi(MINV)\gamma(.)\varepsilon(.)p(.)$	-243.09	6.07	-1.40	3.02	0
$\varphi(TPROD)\gamma(.)\varepsilon(Graz)p(.)$	-243.39	6.49	-1.70	2.06	0
$\varphi(MINV)\gamma(NDVI)\varepsilon(.)p(.)$	-243.50	7.37	-1.81	2.61	0
$\varphi(.)\gamma(NDVI)\varepsilon(.)p(.)$	-243.66	5.95	-1.97	2.29	0
$\varphi(TPROD)\gamma(.)\varepsilon(.)p(.)$	-243.99	6.19	-2.30	2.01	0
$\varphi(.)\gamma(.)\varepsilon(Graz)p(.)$	-244.26	5.41	-2.57	3.11	0
$\varphi(TPROD)\gamma(.)\varepsilon(ForInterv)p(.)$	-244.32	7.06	-2.64	2.04	0
$\varphi(D\_Rip)\gamma(NDVI)\varepsilon(.)p(.)$	-244.42	7.27	-2.73	2.93	0
$\varphi(D\_Rip)\gamma(.)\varepsilon(Graz)p(.)$	-244.69	6.47	-3.00	3.73	0.21
$\varphi(.)\gamma(.)\varepsilon(.)p(.)$	-244.97	5.09	-3.28	2.94	0
$\varphi(.)\gamma(.)\varepsilon(ForInterv)p(.)$	-245.29	5.98	-3.60	2.88	0
$\varphi(D\_Rip)\gamma(.)\varepsilon(.)p(.)$	-245.40	6.19	-3.71	3.50	0
$\varphi(D\_Rip)\gamma(.)\varepsilon(ForInterv)p(.)$	-245.71	7.07	-4.02	3.36	0

* K refers to the effective number of parameters
* ΔSE is the standard error for elpd differences
* Weight is calculated through the stacking method to find the optimal model combination for maximizing the leave-one-out predictive density of the combination distribution. Used for model averaging

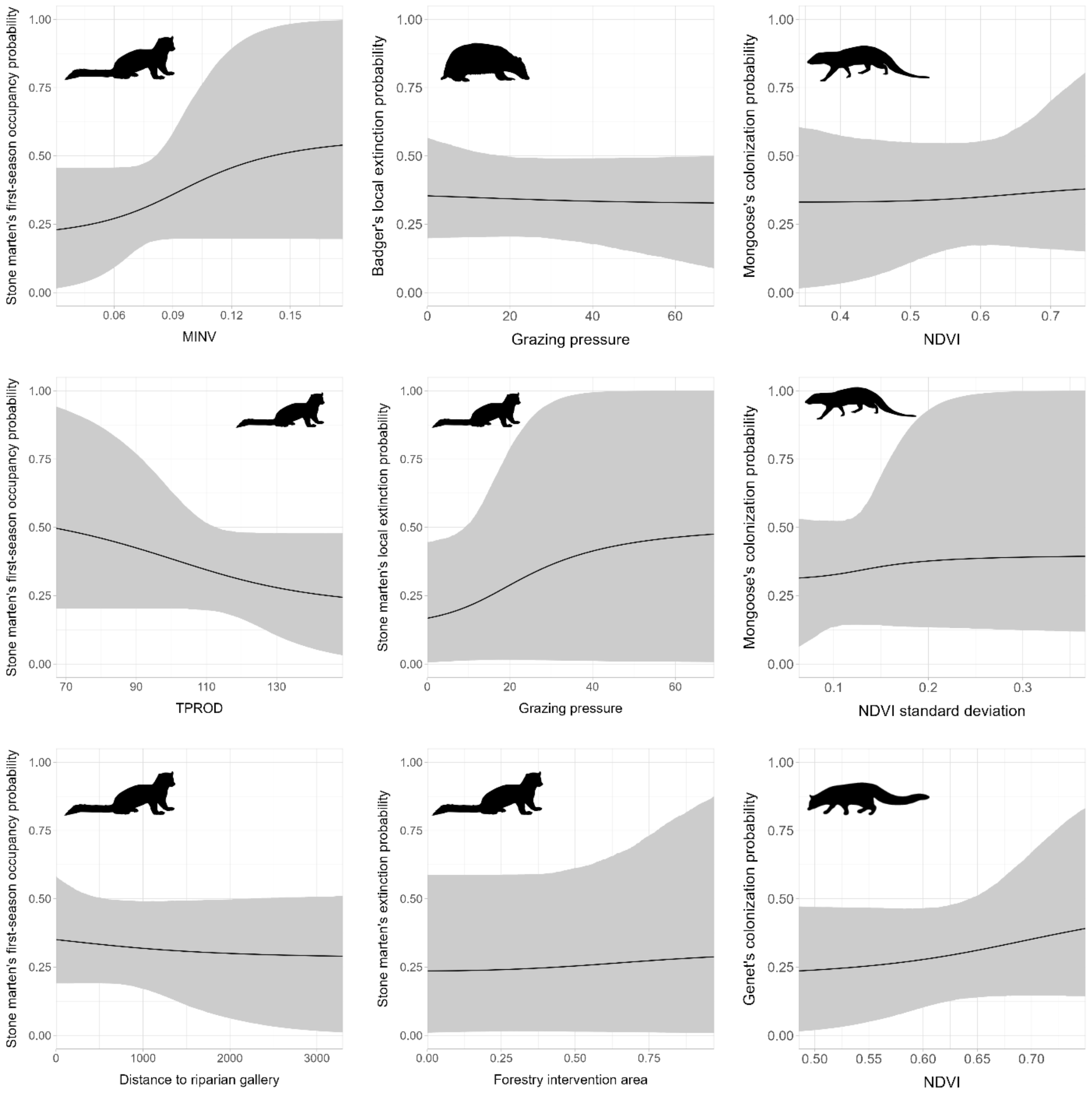

Fig. B1. Covariate effect on state parameters ($\varphi, \gamma, \varepsilon$) for each of the five mesocarnivore species detected at Companhia das Lezírias. Effect plotted only for covariates with a non-significant statistical effect (BCI crosses zero). Shaded area represents 95% BCI. Red fox image from Anthony Caravaggi and used under license CC BY-NC-SA 3.0 DEED (https://creativecommons.org/licenses/by-nc-sa/3.0/). Other species images dedicated to the public domain under license CC0 1.0 DEED.
